# Extracorporeal cardiopulmonary resuscitation: lifesaving for the right patient, at the right time and in the right place

**DOI:** 10.1051/ject/2023043

**Published:** 2023-12-15

**Authors:** Patrick W. Weerwind, Nousjka P.A. Vranken

**Affiliations:** 1 Dept. of Cardiology, Maastricht University Medical Center Maastricht the Netherlands

Prolonged extracorporeal cardiopulmonary support is a medical technology that exemplifies one of the most substantial medical advances in life-saving modalities for patients with cardiorespiratory failure refractory to conventional treatment. This technology is referred to as extracorporeal membrane oxygenation (ECMO) or extracorporeal life support (ECLS). Driven by the success of ECMO in treating acute respiratory patients with H1N1 influenza during the global pandemic in 2009, the use of ECLS has skyrocketed worldwide for adult patients with both respiratory failure and cardiac failure [1]. However, the fastest-growing application of ECLS in the last several years is extracorporeal cardiopulmonary resuscitation (ECPR) as an additional link in the chain of survival in patients with refractory cardiac arrest [2]. It is a complementary intervention when return of spontaneous circulation is not obtained within a reasonable timeframe despite high-quality conventional cardiopulmonary resuscitation (cCPR). ECPR restores and maintains circulation, while buying time for clinicians to identify and potentially reverse the etiology of the event, for example by coronary angiography and subsequent intervention.

Safety and effectiveness of ECPR remain unclear as most data are derived from case series, single-center studies or inconclusive results from randomized clinical trials. Furthermore, the most recent European Resuscitation Council guidelines characterized the evidence supporting ECPR as being very low [3]. Although the first documentation of successful ECPR originated from 1966, mortality following ECPR in adults remains high, over 70% [4, 5]. Undoubtedly, this poor prognosis is related to manifest end-organ ischemia despite ECPR.

Recent reports demonstrate survival to hospital discharge with favorable neurologic outcome in a subset of patients [6, 7]. In these reports, factors to take into account when assessing suitability of ECPR were described. For example, a Japanese study examining data from 120 patients suggests that the first detected heart rhythm is an important determinant of neurologically intact survival, favoring ventricular fibrillation and tachycardia over pulseless electric activity and asystole [8]. Unfortunately, due to the lack of large randomized trials, as well as profound heterogeneity in patient and study characteristics, a robust algorithm to help timely identification of suitable candidates for ECPR is not yet available.

In this issue of the *JECT*, Gutiérrez-Soriano et al. [9] and Michalakes et al. [10] share their experiences with positive and negative outcomes following ECPR for in-hospital and out of hospital cardiac arrest cases. Both case reports highlight key selection criteria and features of developing an ECPR program, emphasizing that improving the outcome of ECPR is multifactorial, with the most identifiable factors being the development of ECLS teams, the optimization of advanced cardiovascular life support and ECPR workflow, and the experience of healthcare providers. Furthermore, to improve ECPR outcome, timing is a crucial aspect i.e., limiting the no-flow time (without CPR) and low-flow time (with cCPR) [11]. Only when circulation is restored, further oxygen debt accumulation is prevented, while sufficient flow is necessary to enable repayment of oxygen debt [12]. In other words, the key factor to ECPR success is minimization of the time to oxygen debt resolution.

Along with the expected metabolic and coagulopathic derangements, ECLS treatment is inherently associated with a high rate of complications such as bleeding, neurologic issues, and infection [2]. Clinicians should also take reperfusion injury into account, particularly cerebral, that may need mitigation [2]. Moreover, successful initiation and weaning from ECLS does not equal survival to hospital discharge, as patients remain susceptible to complications related to the underlying pathology or the received treatment.

Finally, ECPR is a highly complex salvage therapy for when initial cCPR fails in selected cardiac arrest patients. Indeed, ECPR is a multidisciplinary intervention that requires significant resources and training, which are not universally available. While clinicians are challenged to assess the recovery potential of a particular patient i.e., the presumed reversibility of circulatory failure, prolonged decision time will lower the chances of survival drastically. Future studies should aim at unraveling the efficacy of ECPR and factors associated with acceptable neurocognitive outcomes.
